# Placenta Prævia; Treatment by the Caoutchouc Water Pessary

**Published:** 1861-04

**Authors:** E. J. Fountain

**Affiliations:** Davenport, Iowa


### *Placenta Prævia; Treatment by the Caoutchouc Water Pessary.* BY E. J. FOUNTAIN, M. D., Davenport, Iowa.

In view of the fact that in this form of abnormal labor, about one-third of the mothers and two-thirds of the children are lost, it is eminently the duty of every practitioner of the obstetric branch of medical science to labor for the diminution of this fearful mortality, and to record every form of treatment which holds out any promise of improvement. The simplicity and safety of the treatment practised in the following case, and the success attending its application, are sufficient recommendations in its favor to warrant its further trial.

Mrs. P——, aged 20, began suddenly to have uterine hemorrhage, Nov. 26, when seven and a half months advanced in her first pregnancy. No labor-pains or dilatation of the os. The flowing partially ceased under the influence of rest, cold-water enemas, and occasional doses of opium and acetate of lead. In this manner the hemorrhage was kept in check, though recurring to some extent every few days, for two weeks. During this time placenta prævia was suspected, but the os had not dilated sufficiently to render the diagnosis certain.

*Dec.* 9.—Hemorrhage suddenly recurred with great violence, attended with some pain. I now found the os sufficiently dilated to permit the entrance of a finger which came into immediate contact with a placenta. The alarming and dangerous nature of the case was now perfectly apparent. The hemorrhage was profuse, calling for immediate arrest by some other means than the operation of turning, which the rigid and undilated os would not permit. With the view of checking the hemorrhage until the time should arrive when turning might be properly resorted to, I introduced a caoutchouc bag to which was appended a stem by which it could be distended with air or water. At the extremity of this I had a small stop-cock so arranged as to be readily connected with the flexible pipe of Davidson's self-supplying rubber syringe. Dropping the supplying stem of this into a vessel of cold water, a few compressions of the elastic ball quickly filled the bag within the vagina, and at once arrested the hemorrhage. In about half an hour, or perhaps less, a moderate discharge of blood again appeared, forcing its way around the pessary. I immediately allowed the water, now quite warm, to escape without withdrawing the instrument, and quickly refilled it with cold water, when all flowing again ceased. This proceeding was continued through the day and following night, the warm water being frequently discharged and replaced by cold; and so certain was this in its effect that I left the patient several times for an hour or two to attend to other urgent calls, the husband in the meantime refilling the ball with cold water as often as any symptom of hemorrhage appeared. Occasionally, when changing the contents from warm to cold water, I would make an examination. This could be effected without even removing the instrument. When this treatment had been continued about twenty-four hours I found the os becoming well dilated and labor pains more regular. The margin of the placenta could not be reached, nor was any part of the child presenting. I now felt satisfied the time had arrived when I could safely accomplish the operation of turning, but the efficiency of the vaginal bag of water in safely conducting the case to this period began to impress me with the belief that its continuance might obviate the necessity of turning. At all events, I reasoned, it can do no harm to try it, being ready at any moment to resort to the latter operation, for which the parts were becoming all the time more favorable. From this time the pains steadily increased, and within an hour I had the gratification of feeling a presenting vertex crowding upon one side of the placenta. After this the disposition to hemorrhage gradually ceased. As soon as the advancing vertex had partially passed between the placenta and now widely dilated margin of the open cervix, I removed the pessary permanently. The labor now progressed favorably without further hemorrhage, and the child, a female, was born living and in good condition, about thirty hours after the commencement of the above described treatment. The placenta was found loose in the vagina immediately after the delivery of the child.

In the recent and very instructive lecture on placenta prævia by T. Gaillard Thomas, M. D., the pathological character of this complication of labor and indications in methods of treatment are briefly stated with that vigor and terseness of language and philosophical reasoning which characterize all the productions of his pen with which he has favored the profession. In cases of the kind above reported, where the os is rigid and the hemorrhage profuse, he recommends a tampon of sponge saturated with the solution of the perchloride of iron, and, "Better still than a tampon, the instrument called the colpeurynter might be used, or in place of it a hog's bladder, tied to the end of a self-supplying syringe, introduced in a collapsed state into the vagina and then filled with water, may be employed. But remember, this is only temporizing, and that it merely prepares the way for the fulfilment of an important indication which it by no means effects itself."

It was with this view that I resorted to such a mode of treatment, fully expecting to be obliged to follow it up by the operation of turning. I soon found that it could not be depended upon as a tampon simply, but the facility with which it permitted of a constant application of cold enabled me, by its influence in connexion with the pressure, to control the hemorrhage effectually, not merely as a temporizing expedient but permanently, and with the important result of saving the child as well as mother. This does not by any means invalidate the correctness of the principles advocated by Dr. Thomas, but certainly establishes an exception to the general rule. How frequently success may attend the treatment can of course be determined only by further trial. Its great superiority to any other form of tampon is readily apparent, and it is applicable to all cases where a tampon is required. I can speak from experience, of efficacy in cases of flooding connected with abortion. A peculiar feature of the treatment is that it readily admits of a constant application of *cold* in connexion with the best form of mechanical *pressure.* The latter alone I found to be insufficient, but in connection with the former, every indication was fully answered; and thus my patient escaped the danger of a serious operation, and the child (which is living and doing well) is undoubtedly indebted to the same for the preservation of its life.—*American Medical Times.*

